# The Challenge of Type 3c Diabetes: From Accurate Diagnosis to Effective Treatment

**DOI:** 10.1210/jcemcr/luaf109

**Published:** 2025-05-21

**Authors:** Ilaria Milani, Gloria Guarisco, Marianna Chinucci, Chiara Gaita, Frida Leonetti, Danila Capoccia

**Affiliations:** Faculty of Pharmacy and Medicine, Department of Medico-Surgical Sciences and Biotechnologies, Sapienza University of Rome, Latin 04100, Italy; Faculty of Pharmacy and Medicine, Department of Medico-Surgical Sciences and Biotechnologies, Sapienza University of Rome, Latin 04100, Italy; Faculty of Pharmacy and Medicine, Department of Medico-Surgical Sciences and Biotechnologies, Sapienza University of Rome, Latin 04100, Italy; Faculty of Pharmacy and Medicine, Department of Medico-Surgical Sciences and Biotechnologies, Sapienza University of Rome, Latin 04100, Italy; Faculty of Pharmacy and Medicine, Department of Medico-Surgical Sciences and Biotechnologies, Sapienza University of Rome, Latin 04100, Italy; Faculty of Pharmacy and Medicine, Department of Medico-Surgical Sciences and Biotechnologies, Sapienza University of Rome, Latin 04100, Italy

**Keywords:** type 3c diabetes mellitus, chronic pancreatitis, glucose monitoring, diagnosis, treatment

## Abstract

Type 3c diabetes mellitus is a secondary form of diabetes associated with pancreatic disease, primarily chronic pancreatitis, which impairs insulin and glucagon secretion, resulting in inadequate glycemic control. Often misdiagnosed as other types of diabetes, such as type 1 or type 2 diabetes mellitus, type 3c diabetes mellitus is characterized by significant glucose variability, increased insulin requirements, and risk of hypoglycemia.

This case report describes a 24-year-old man with a history of hereditary chronic pancreatitis resulting from a serine protease 1 (*PRSS1*) gene pathogenic variant, who presented with fasting hyperglycemia and elevated glycated hemoglobin requiring early insulin therapy. In the absence of symptoms of exocrine pancreatic insufficiency, specific diagnostic criteria confirmed the diagnosis of pancreatogenic diabetes, and the use of a glucose monitoring system proved essential for optimal management. Therefore, appropriate screening for diabetes in patients with hereditary chronic pancreatitis, combined with accurate diagnosis and close monitoring, can lead to the development of individualized strategies to prevent complications and improve glycemic control.

## Introduction

Type 3c diabetes mellitus (T3cDM) is a distinct form of diabetes recognized by both the American Diabetes Association and the World Health Organization [1]. Known as “pancreatogenic or pancreoprivic,” it is a secondary form of diabetes associated with diseases of the exocrine pancreas [2], with chronic pancreatitis (CP) being the primary cause (79%), followed by pancreatic ductal adenocarcinoma (8%), hemochromatosis (7%), cystic fibrosis (4%), and pancreatic surgery (2%) [3]. Genetic pathogenic variants are strongly associated with the development of pancreatic diseases. The c.311T > C (p.L104P) pathogenic variant of the serine protease 1 (*PRSS1*) gene (encoding cationic trypsinogen) is frequently found in families with hereditary chronic pancreatitis (HCP) [4, 5], often presents as recurrent acute pancreatitis and serves as precursor to CP [1, 6, 7]. Genetic testing is used to diagnose this familial disorder that predisposes to complications such as T3cDM. Approximately 48% of individuals with *PRSS1*-associated HCP will develop diabetes, a prevalence similar to other forms of CP [8].

T3cDM is difficult to diagnose, leading to underdiagnosis [1]. It affects approximately 5% to 10% of people with diabetes but is often misclassified as type 1 (T1DM) or type 2 (T2DM) diabetes mellitus [9] its unique clinical and metabolic features [10]. To improve diagnosis, Ewald and Bretzel proposed specific diagnostic criteria [11, 12], including testing for exocrine pancreatic insufficiency (fecal monoclonal elastase-1 test or direct functional testing), pancreatic imaging (endoscopic ultrasound, magnetic resonance imaging, and computed tomography), and the absence of autoimmune markers typical of T1DM [13]. Assessment of β-cell function (eg, C-peptide levels) or low levels of lipid-soluble vitamins (A, D, E, K) are additional criteria [14].

These criteria are not yet standardized and can be difficult to apply, especially in cases that overlap with T1DM or long-standing T2DM complicated by exocrine pancreatic insufficiency [2]; however, their early adoption can improve the detection and management of T3cDM [9, 13]. The unique pathophysiology of T3cDM, which includes pancreatic inflammation, fibrosis, and loss of insulin-, glucagon-, and pancreatic polypeptide-secreting cells, requires tailored treatment strategies [2, 15].

The refinement and use of these diagnostic criteria is essential for patients who may not have typical signs of exocrine pancreatic insufficiency but who could benefit from early insulin therapy and close monitoring.

## Case Presentation

At the first visit, a 24-year-old man was referred for evaluation of fasting hyperglycemia and elevated glycated hemoglobin (HbA1c) (Table 1). He was overweight (body mass index = 27.8 kg/m^2^), had normal blood pressure (130/80 mm Hg), and reported no acute symptoms of hyperglycemia. The patient had a family history of CP, with his mother developing diabetes, probably secondary to pancreatitis. From 2014 to 2019, he had multiple documented episodes of acute pancreatitis with elevated amylase and lipase levels. Genetic testing revealed a heterozygous pathogenic variant in the *PRSS1* gene (c.311T > C, p.L104P), which was also found in several family members. Additional testing for other pancreatitis risk genes (*SPINK1, PRSS2, CTRC, CASR, CTSB, KRT8*, and *CPA1*) were negative. A magnetic resonance imaging scan and computed tomography scan in 2020 showed pancreatic atrophy, especially in the body, and chronic inflammation with a tortuous common bile duct. Since 2020, he has had no further episodes of acute pancreatitis. Pancreatic enzyme replacement therapy and proton pump inhibitors were recommended during his hospitalizations.

**Table 1.** Anthropometric and biochemical test at first visit and during follow-up

Anthropometric and biochemical test	First visit	3 months	6 months	9 months	12 months	Normal reference range
BMI	27.8 kg/m^2^	29 kg/m^2^	28.7 kg/m^2^	28.7 kg/m^2^	28.8 kg/m^2^	18.5-24.9 kg/m^2^
Glycemia	189 mg/dL (10.5 mmol/L)	170 mg/dL (9.44 mmol/L)	110 mg/dL (6.11 mmol/L)	119 mg/dL (6.61 mmol/L)	112 mg/dL (6.22 mmol/L)	70-105 mg/dL (3.89-5.83 mmol/L)
Glycated hemoglobin	9.3% (78 mmol/mol)	8.9% (74 mmol/mol)	7.6% (60 mmol/mol)	8.1% (65 mmol/mol)	6.9% (52 mmol/mol)	4.3-5.9% (20-42 mmol/mol)
C-peptide	0.8 ng/mL (0.26 nmol/L)	—	—	—	—	0.8-3.1 ng/mL (0.26-1.03 nmol/L)
Total cholesterol	208 mg/dL (5.37 mmol/L)	218 mg/dL (5.63 mmol/L)	168 mg/dL (20-42 mmol/L)	206 mg/dL (4.34 mmol/L)	156 mg/dL (4.03 mmol/L)	<200 mg/dL (5.17 mmol/L)
LDL cholesterol	146 mg/dL (3.77 mmol/L)	156 mg/dL (4.03 mmol/L)	109 mg/dL (2.82 mmol/L)	148.4 mg/dL (3.83 mmol/L)	102.2 mg/dL (2.64 mmol/L)	<100 mg/dL (2.6 mmol/L)
HDL cholesterol	42 mg/dL (1.08 mmol/L)	42 mg/dL (1.08 mmol/L)	44 mg/dL (1.13 mmol/L)	46 mg/dL (1.18 mmol/L)	43 mg/dL (1.11 mmol/L)	>45 mg/dL (1.16 mmol/L)
Triglycerides	98 mg/dL (1.10 mmol/L)	100 mg/dL (1.12 mmol/L)	72 mg/dL (0.81 mmol/L)	58 mg/dL (0.65 mmol/L)	54 mg/dL (0.61 mmol/L)	<150 mg/dL (1.69 mmol/L)
Creatinine	0.83 mg/dL (0.07 mmol/L)	0.89 mg/dL (0.08 mmol/L)	1.02 mg/dL (0.09 mmol/L)	1.17 mg/dL (0.10 mmol/L)	1.08 mg/dL (0.09 mmol/L)	0.73-1.18 mg/dL (0.06-0.104 mmol/L)
eGFR MDRD	118 mL/min	108 mL/min	93 mL/min	74 mL/min	85.92 mL/min	>60 mL/min
GOT	64 U/L	—	—	18 U/L	—	5-34 U/L
GPT	38 U/L	—	—	37 U/L	—	0-55 U/L
Amylase	36 U/L	—	—	6 U/L	—	28-100 U/L
Lipase	11 U/L	—	—	7 U/L	—	<59 U/L
Fecal elastase	200 mcg/g	—	—	—	—	>200 mcg/g

Abbreviations: BMI, body mass index; eGFR, estimated glomerular filtration rate; GOT = glutamic oxaloacetic transaminase; GPT = glutamic pyruvic transaminase; MDRD, Modification of Diet in Renal Disease.

## Diagnostic Assessment

At the first visit, given the uncontrolled glycemic control of the patient, the medical history of HCP and the young age, further testing was required according to the Ewald and Bretzel criteria to differentiate the diagnosis. These tests included T1DM autoantibodies, fecal elastase 1 levels, pancreatic imaging, and plasma C-peptide levels to assess β-cell function. Because of his high HbA1c, insulin glargine U 300 (10 IU) and metformin (500 mg twice daily) were prescribed.

Over the next year, the patient did not report any gastrointestinal symptoms of exocrine pancreatic insufficiency and therefore did not adhere to the recommended pancreatic enzyme replacement therapy and proton pump inhibitor therapy or attend a specialized outpatient clinic.

At a 3-month follow-up, laboratory test confirmed a second high glucose and HbA1c value (Table 2) according to the American Diabetes Association criteria for diabetes [12]. Autoimmune markers for T1DM (anti-GAD, anti-ICA, and anti-Znt8) were negative, but C-peptide fasting levels indicated reduced β-cell function (Table 1). Pancreatic imaging (endoscopic ultrasound) showed heterogeneous and atrophic pancreatic parenchyma, hyperechoic clots in the lumen, and dilated main (9 mm) and accessory pancreatic ducts without evidence of cancer. A diagnosis of HCP-related T3cDM was made.

**Table 2.** Self-blood glucose monitoring

	Before breakfast	2 hours after breakfast	Before lunch	2 hours after lunch	Before dinner	2 hours after dinner
			124 mg/dL (6.8 mmol/L)	117 mg/dL (6.5 mmol/L)		
			125 mg/dL (6.9 mmol/L)	96 mg/dL (5.3 mmol/L)		
	139 mg/dL (7.7 mmol/L)	127 mg/dL (7.0 mmol/L)				
	121 mg/dL (6.7 mmol/L)	101 mg/dL (5.6 mmol/L)				
			117 mg/dL (6.5 mmol/L)		90 mg/dL (5 mmol/L)	
					91 mg/dL (5.05 mmol/L)	108 mg/dL (6 mmol/L)
	132 mg/dL (7.3 mmol/L)	114 mg/dL (6.3 mmol/L)				
			130 mg/dL (7.2 mmol/L)	98 mg/dL (5.4 mmol/L)		
					100 mg/dL (5.5 mmol/L)	115 mg/dL (6.4 mmol/L)
			87 mg/dL (4.8 mmol/L)		90 mg/dL (5 mmol/L)	
			107 mg/dL (5.9 mmol/L)	79 mg/dL (4.4 mmol/L)		
	121 mg/dL (6.7 mmol/L)	103 mg/dL (5.7 mmol/L)				
			89 mg/dL (4.9 mmol/L)	88 mg/dL (4.8 mmol/L)		
					102 mg/dL (5.6 mmol/L)	119 mg/dL (6.6 mmol/L)
	127 mg/dL (7.0 mmol/L)	108 mg/dL (6 mmol/L)				
			123 mg/dL (6.8 mmol/L)	99 mg/dL (5.5 mmol/L)		
			104 mg/dL (5.8 mmol/L)	97 mg/dL (5.4 mmol/L)		
					105 mg/dL (5.8 mmol/L)	89 mg/dL (4.9 mmol/L)
	95 mg/dL (5.3 mmol/L)	103 mg/dL (5.7 mmol/L)				
			87 mg/dL (4.8 mmol/L)	68 mg/dL (3.7 mmol/L)		
			105 mg/dL (5.8 mmol/L)	77 mg/dL (4.3 mmol/L)		
					105 mg/dL (5.8 mmol/L)	98 mg/dL (5.4 mmol/L)
Normal range	70-130 mg/dL (3.9-7.2 mmol/L)	< 180 mg/dL (< 10 mmol/L)	70-130 mg/dL (3.9-7.2 mmol/L)	< 180 mg/dL (< 10 mmol/L)	70-130 mg/dL (3.9-7.2 mmol/L)	< 180 mg/dL (< 10 mmol/L)

## Treatment

Because of suboptimal glycemic control and the absence of hypoglycemic events, insulin glargine U 300 was increased to 16 IU and metformin was increased to 1000 mg twice daily. Lipid-lowering therapy was also prescribed for elevated low-density lipoprotein (LDL) cholesterol levels.

At the 6-month follow-up visit, the patient's HbA1c and fasting glucose levels improved (Table 1). However, the patient declined the use of continuous glucose monitoring (CGM), opting instead for self-reported blood glucose monitoring (SBGM).

**Figure 1.**
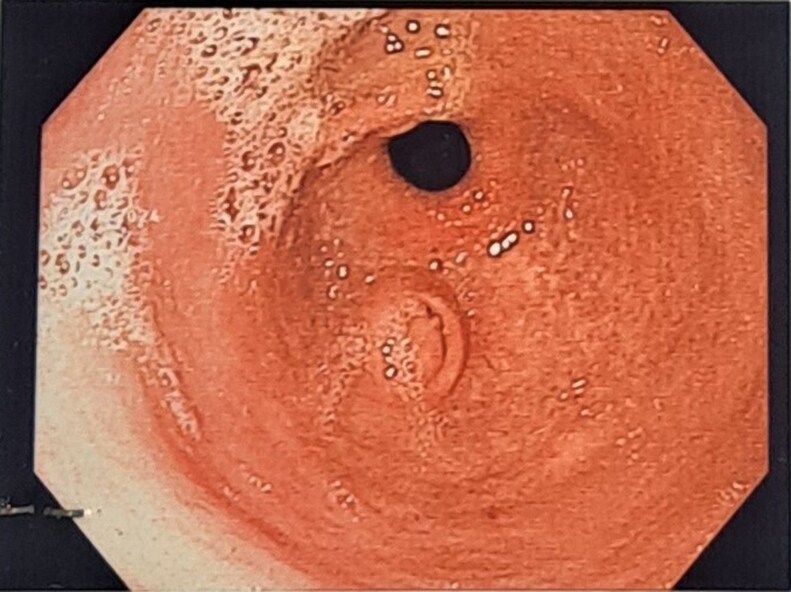
Esophagogastroduodenoscopy revealing minute swelling with central umbilication located in the gastric antrum, as for aberrant pancreas.

Despite the previous improvement in glycemic control, laboratory results at the 9-month follow-up visit (Table 1) showed an increase in HbA1c. This was in contrast to the low fasting glucose and trend toward lower glucose levels observed in the SBGM (Table 2). A capillary blood glucose test 3 hours after a meal was 107 mg/dL (5.9 mmol/L)(normal range: 70-130 mg/dL; 3.8-7.2 mmol/L), highlighting discrepancies with HbA1c. In addition, there was low pancreatic enzyme activity (lipase and amylase) but normal liver function and no evidence of anemia or blood cell changes (Table 1). The patient agreed to start using CGM and, given the elevated LDL cholesterol levels, was instructed to improve adherence to prescribed lipid-lowering therapy. An esophagogastroduodenoscopy revealed a small swelling with central umbilication in the lower wall of the gastric antrum, suggesting an aberrant pancreas (Fig. 1).

## Outcome and Follow-up

After 3 weeks, analysis of the CGM data showed positive glycemic control with 80% time in range, 20% time above range, 0% time below range, and 6.8% of estimated HbA1c. The coefficient of variation was 32%. Because of occasional postprandial spikes, therapy was intensified by adding rapid-acting insulin (5 IU before breakfast, 4 IU before lunch, and 4 IU before dinner), and the patient was instructed on how to adjust the insulin dose as needed. LDL cholesterol levels improved.

**Figure 2.**
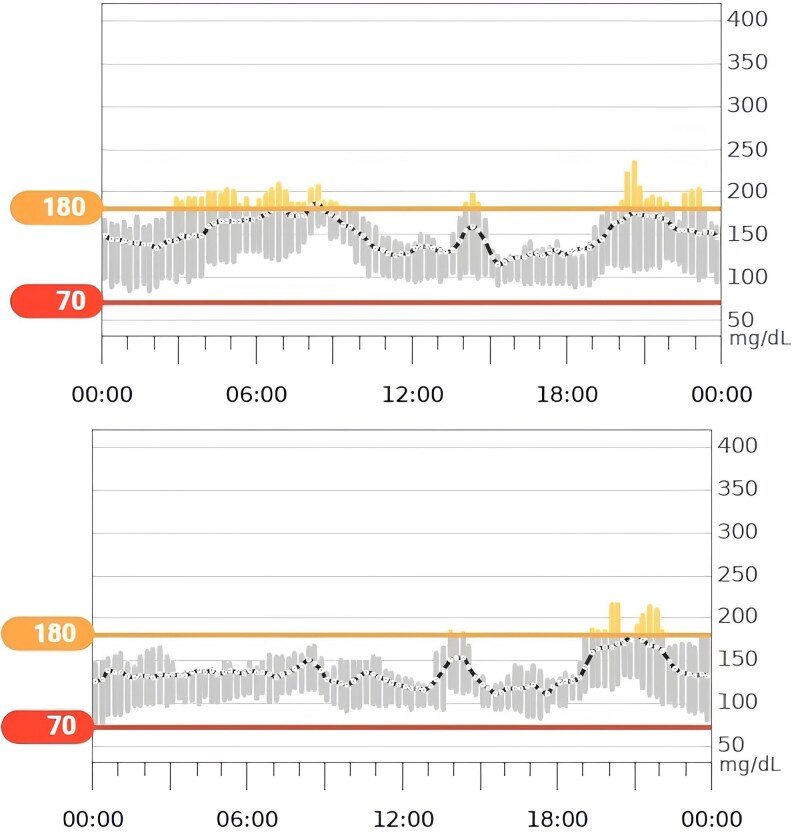
Patient's CGM change before and after treatment.

After an additional 3 weeks, CGM data confirmed that good glycemic control was maintained (Fig. 2), achieving a good glycemic and lipid control at 12 months (Table 1).

## Discussion

This case report emphasizes the importance of suspecting T3cDM in patients with a history of HCP or CP [16], especially those with *PRSS1* gene pathogenic variant, which typically result in the *PRSS1* histopathologic imaging triad: pancreatic atrophy, calcification, and ductal dilatation [17], as seen in the patient. Research shows that approximately 80% of individuals with *PRSS1* pathogenic variant have diffuse pancreatic atrophy and 60% have ductal dilatation (6-22 mm). In addition, replacement of pancreatic tissue by adipose tissue has been observed, leading to lipomatous atrophy [5], which increases the risk of pancreatic cancer [18]. This highlights the need for regular cancer screening during follow-up [3].

T3cDM, often called “brittle diabetes,” is characterized by fluctuating blood glucose levels because of both insulin resistance and insulin deficiency [2, 9, 15]. Currently, there are no standardized treatment guidelines for T3cDM; this can lead to undertreatment or unsafe management [19]. Expert consensus [20] recommends tight glycemic control (HbA1c < 7%) to prevent complications [1].

First-line treatment includes metformin and insulin [14], especially in cases with HCP [9], tailored to the patient's clinical presentation [14]. Metformin is effective for mild hyperglycemia (HbA1c < 8%) and insulin resistance [21], and may protect against pancreatic cancer [14], a common complication in these patients. However, gastrointestinal side effects can be a limitation [21]. Many patients often require intensified therapy [11, 19, 21], including early insulin initiation [22], recommended by consensus guidelines for the treatment of insulin deficiency in diabetes secondary to CP [20]. Insulin should be started gradually to avoid hypoglycemia, but higher doses may be needed if insulin resistance is suspected [22]. Experts recommend continuing metformin and other oral agents to reduce insulin doses [10, 19], while close monitoring is essential [11].

The role of other antidiabetic agents remains uncertain [14, 19]. Thiazolidinediones carry risks such as bone fractures and fluid retention, whereas sulfonylureas increase the risk of severe hypoglycemia. Incretin-based therapies (glucagon-like peptide-1 receptor agonists and oral dipeptidyl peptidase-4 inhibitors) are avoided because of concerns about pancreatitis. Sodium-glucose cotransporter-2 inhibitors are not recommended for T3cDM until their safety is confirmed with regard to the risk of euglycemic diabetic ketoacidosis in insulin-deficient states [14, 21].

The increased fragility of T3cDM is often associated with a high risk of hypoglycemia. However, in clinical practice, inadequate pancreatic β-cell function and insulin resistance also contribute to higher insulin requirements and poorer glycemic control, particularly in terms of hyperglycemic events [14]. This may explain the higher HbA1c levels in patients with T3cDM compared to those with T2DM [23], as in this patient. Additionally, HbA1c does not accurately reflect daily glucose fluctuations [24], which may not correlate with estimated HbA1c from CGM [25] or SBGM, as shown in this case report.

Studies using CGM suggest that patients with T3cDM are likely to experience more hyperglycemic episodes. Higher glucose variability (GV) was found in patients with fibrocalcific pancreatic diabetes compared to those with T2DM, mainly attributed to postprandial glucose spikes [26]. Similarly, time above range was found to be higher in totally pancreatectomized patients than in patients with T1DM, despite similar GV [27]. Recent research has shown that patients with T3cDM have higher estimated HbA1c and blood glucose levels, but do not have higher GV compared to those with T1DM and T2DM. In addition, patients with T3cDM had lower C-peptide levels than patients with T2DM, but higher than those with T1DM [28]. These studies did not observe an increased risk of hypoglycemia in T3cDM.

These findings suggest that although HbA1c levels may not accurately reflect glycemic fluctuations, CGM may provide more accurate management [26, 28] and optimize glycemic control in these patients. As demonstrated in this case, despite a stepwise intensification of insulin therapy, the introduction of prandial insulin guided by CGM improved glycemia due to the patient's minimal residual β-cell function and likely contributed to the improvement in the trend toward lower glycemia observed in the SBGM. This underscores the importance of CGM in improving glycemic control and reducing GV, as evidenced by coefficient of variation <36%, which identifies stable vs unstable glycemia [24]. In addition, close patient monitoring allows for a more personalized approach to treatment, with strategies tailored to the specific needs of patients with T3cDM. Although insulin is the most commonly recommended therapy for T3cDM [11], further research using continuous monitoring may explore a broader range of hypoglycemic treatment options once better glycemic control is achieved.

## Learning Points

- T3cDM has unique features that make its diagnosis and management complex and require careful attention from health care providers.
- HCP, often associated with the *PRSS1* pathogenic variant, is a primary cause of CP, which can lead to the development of T3cDM.
- Given the atypical presentation of T3cDM, it is important to increase awareness and screen for diabetes in patients with CP or HCP, even in the absence of severe exocrine insufficiency symptoms.
- Accurate diagnosis of T3cDM requires specific criteria to distinguish it from other forms of diabetes.
- Early detection of T3cDM is critical for timely treatment, with close monitoring and the use of technology potentially improving patient outcomes and enabling the exploration of new treatment strategies.

## Contributors

All authors made individual contributions to authorship. D.C. and M.C. were involved in the diagnosis and management of the patient and in the manuscript design, G.G. and C.G. collected the data, and I.M. contributed to the original draft writing and prepared the tables and figures. F.L. revised the manuscript and D.C. supervised the manuscript submission. All authors reviewed and approved the final draft.

## Data Availability

Data sharing is not applicable to this article as no data sets were generated or analyzed during the present study.
